# Longitudinal Analysis of Music Education on Executive Functions in Primary School Children

**DOI:** 10.3389/fnins.2018.00103

**Published:** 2018-02-28

**Authors:** Artur C. Jaschke, Henkjan Honing, Erik J. A. Scherder

**Affiliations:** ^1^Clinical Neuropsychology, VU University Amsterdam, Amsterdam, Netherlands; ^2^Music Therapy, ArtEZ University of the Arts, Enschede, Netherlands; ^3^Music Cognition Group, Amsterdam Brain and Cognition, Institute for Logic, Language, and Computation, University of Amsterdam, Amsterdam, Netherlands

**Keywords:** longitudinal analysis, music, intelligence, executive functions, far transfer

## Abstract

**Background:** Research on the effects of music education on cognitive abilities has generated increasing interest across the scientific community. Nonetheless, longitudinal studies investigating the effects of structured music education on cognitive sub-functions are still rare. Prime candidates for investigating a relationship between academic achievement and music education appear to be executive functions such as planning, working memory, and inhibition.

**Methods:** One hundred and forty-seven primary school children, M_age_ = 6.4 years, *SD* = 0.65 were followed for 2.5 years. Participants were randomized into four groups: two music intervention groups, one active visual arts group, and a no arts control group. Neuropsychological tests assessed verbal intelligence and executive functions. Additionally, a national pupil monitor provided data on academic performance.

**Results:** Children in the visual arts group perform better on visuospatial memory tasks as compared to the three other conditions. However, the test scores on inhibition, planning and verbal intelligence increased significantly in the two music groups over time as compared to the visual art and no arts controls. Mediation analysis with executive functions and verbal IQ as mediator for academic performance have shown a possible far transfer effect from executive sub-function to academic performance scores.

**Discussion:** The present results indicate a positive influence of long-term music education on cognitive abilities such as inhibition and planning. Of note, following a two-and-a-half year long visual arts program significantly improves scores on a visuospatial memory task. All results combined, this study supports a far transfer effect from music education to academic achievement mediated by executive sub-functions.

## Introduction

Arguing in favor of far transfer from music lessons to academic achievement remains difficult (Ho et al., 2003; Costa-Giomi, 2004; Schellenberg, 2006; Degé et al., 2011; Moreno et al., 2011; Tsang and Conrad, 2011; Rodrigues et al., 2013; Roden et al., 2014; Dumont et al., 2017; Holochwost et al., 2017). Researchers comparing musical with non-musical groups have concluded that personality traits, as well as economic status, can contribute to the reason participants take up music training, implying possible advantages in cognitive functions and therefore motivation to follow music lessons (Corrigall et al., 2013). Analyzing far transfer in randomized controlled longitudinal studies however, has minimized factors like home support, socioeconomic status, available resources, peer-to-peer interaction, or musical aptitude (Roden et al., 2013). Yet proving a possible far transfer effect from music education to academic achievement remains a difficult task. There seems to be little evidence that musical skills transfer directly to, for example, mathematics or language (Mehr et al., 2013; Dumont et al., 2017; Kraus and White-Schwoch, 2017; Sala and Gobet, 2017). The prime candidate when analyzing a possible far transfer effect from music skills to cognitive functioning and academic achievement appears to be executive functions. The sub-division of executive functions (EF also known as *cognitive control* or *executive control*) such as initiation, planning, attention, inhibition and working memory (Corrigall et al., 2013; Mehr et al., 2013; Roden et al., 2013; Sala and Gobet, 2017) play a crucial role in general cognitive processes (Engle, 2002; Slevc et al., 2016). Researchers have concluded that an increase in intelligence scores and thus academic skills was mediated by higher performances on EF tasks in children receiving music lessons (Degé et al., 2011). These studies, however, have received a fair amount of criticism as the relationship between music, intelligence, and academic skills was not made clear. Reasons for this were found in children being neither matched at baseline nor randomized. This could imply that children with a higher IQ may also have increased executive functioning skills, which might enable them to persist in their music studies thereby benefiting from a possible long-term effect of music on EF. Additionally, umbrella terms were used to describe executive functioning. These did not allow for a structured analysis of related sub-functions such as inhibition, planning or working memory.

Zuk et al. (2015), have investigated the neural correlates of executive functioning in both adult and child musicians and non-musicians with functional magnetic resonance imaging (fMRI) and neuropsychological testing. Their results have shown increased activation in the areas of the brain traditionally associated with EF regions, like the ventro-lateral and the medial prefrontal cortex in child musicians. The authors conclude that a direct connection between neural correlates of EF and musical skills is highly possible given the extended demand of planning, attention, working memory, and inhibition when playing or singing. While investigating this relationship, Roden et al. (2013, 2014) have compared an active music group to a science class group and concluded that the music group increased on auditory working memory capacity over a period of 18 months. The authors stressed however, that these results have to be interpreted with caution as they have used a quasi-experimental design without randomization of participants. Furthermore, they suggest that children receiving music lessons only improve in music related cognitive domains such as auditory working memory, supporting a near but not a far transfer effect. Costa-Giomi (2004) has researched the effect of 3 years of piano lessons on academic achievement and self-esteem in 117 fourth grade children. Even though self-esteem has significantly improved there was no transfer effect to academic achievement in this group. Further, researchers have investigated a possible far transfer effect from music lessons to mathematical abilities in 5-year-old children (Mehr et al., 2013). Children were randomly assigned to either a music program where they sang songs, danced, and played with shakers and/or sticks or to a visual arts group. The authors found no significant difference between both groups on tests associated with mathematical skills, thus no far transfer from music to mathematics (Mehr et al., 2013). Contrary to these findings, a recent study has investigated a possible transfer from music education to academic achievement in a 3-year follow-up in 11 and 14-year-old children (dos Santos-Luiz et al., 2015). Controlling for intelligence, socioeconomic status, and motivation, the authors have found evidence of a far transfer effect from music education to increased Portuguese language skills and marks in natural science. The same study however, has found a weaker transfer relationship with history and geography and transfer was least pronounced to mathematics and English language skills.

In sum, inconclusive results (Detterman, 1993; Halpern, 1998; Barnett and Ceci, 2002; Engle, 2002; Ho et al., 2003; Costa-Giomi, 2004; Schellenberg, 2006; Degé and Schwarzer, 2011; Degé et al., 2011; Moreno et al., 2011; Tsang and Conrad, 2011; Rickard et al., 2012; Corrigall et al., 2013; Mehr et al., 2013; Roden et al., 2013, 2014; Rodrigues et al., 2013; Benz et al., 2015; dos Santos-Luiz et al., 2015; Flaugnacco et al., 2015; Zuk et al., 2015; Slevc et al., 2016; Swaminathan and Schellenberg, 2016; Dumont et al., 2017; Holochwost et al., 2017; Kraus and White-Schwoch, 2017; Sala and Gobet, 2017) often find their origin in being set up as correlational. Investigations using a longitudinal design are often quasi-experimental designs lacking randomization (Roden et al., 2014) or use non-active control groups (Engle, 2002; Ho et al., 2003; Costa-Giomi, 2004; Schellenberg, 2006; Degé et al., 2011; Moreno et al., 2011; Tsang and Conrad, 2011; Rodrigues et al., 2013; Roden et al., 2014; Slevc et al., 2016; Dumont et al., 2017; Holochwost et al., 2017). In light of these ambiguous findings, three recent reviews (Benz et al., 2015; Dumont et al., 2017; Sala and Gobet, 2017) have shown that research into the effects of music interventions on cognitive skills, even though promising, still need more randomized longitudinal studies to support a positive claim.

To address this issue, we investigated the influence of a structured music education program in primary school children with a block randomization longitudinal design. Arts programs were introduced into the school curriculum to reach every student in the participating groups. The music group was compared to an active visual arts control as well as a no arts control group. It is hypothesized therefore that music education will improve EF sub-functions i.e., inhibition, planning, and working memory, thus supporting a far transfer effect to academic achievement. We have chosen these executive sub-functions as they are needed for both academic tasks and learning how to play music or sing (Detterman, 1993; Halpern, 1998; Barnett and Ceci, 2002; Roden et al., 2013; Swaminathan and Schellenberg, 2016; Dumont et al., 2017; Sala and Gobet, 2017).

## Methods

### Design

The design of the present longitudinal study was a block randomization controlled trial with repeated measures across three groups: MUSIC, VISUAL ARTS, and NO ARTS. A fourth group, MUSIC + was added *post-hoc* to our data collection for inclusion into statistical analysis and was therefore not an option in the randomization procedure as described here.

MOCCA, an expert center for creating and applying arts-based and general educational programs selected primary schools across the Netherlands from an extensive database of Dutch primary schools. All 153 schools from this database were assigned identification numbers. These numbers were forwarded to an independent administration worker. S/he has randomly selected six^1^ identification numbers, i.e., schools, to be included into our study using the *RAND* function in Microsoft® Excel. Finally, MOCCA assigned two schools (blocks) to one of the three conditions: two schools to the music intervention, two schools to the visual arts intervention and two schools to the no arts control. Participants followed the regular Dutch school curriculum for primary schools and have received the music or visual arts intervention as supplementary to the regular curriculum^2^. The researchers were blind to the selection procedure.

### Participants

Initially *N* = 230 participants across six primary schools were approached to participate, *N* = 176 were tested at baseline (52.4% girls) with a mean age of *M*_age_ = 6.4 years, *SD* = 0.65. According to Menard (ed.) et al. (Menard, 2007), a 15% drop-out rate for longitudinal designs could be expected due to personal or geographical reasons. Missing values i.e., incomplete test results, sickness of the participants at any testing moment or unexpected termination of the test were excluded from the final data analysis. Throughout all testing moments outliers were identified as any score on any test which could be classified as outside the outer fences of the test as cross checked within the used statistical software^3^.

The baseline sample was divided into four groups and, after accounting for missing values, outliers and drop outs (**Figure 2**), the final analysis was performed over *N* = 147 participants: (1) *MUSIC* + (*N* = 38; *M*_age_ = 6.3, *SD* = 0.52) music intervention with prior music knowledge (private instrumental lessons outside of the regular school curriculum *M* = 3.5 months, *SD* = 0.89 and continued these in parallel with our music intervention); (2) Music intervention no prior Music knowledge *MUSIC* (*N* = 42; *M*_age_ = 6.4, *SD* = 0.44), (3) *VISUAL ARTS* (*N* = 29; *M*_age_ = 6.6, *SD* = 0.48), and (4) *NO ARTS* control (*N* = 37; *M*_age_ = 6.2, *SD* = 0.73).

Exclusion criteria were set at the inability to perform neuropsychological testing due to dyslexia, dyscalculia, severe deafness, and blindness or insufficient motor command of both arms as well as children in either control condition (VISUAL or NO ARTS) who received private music lessons.

Informed consent was obtained from parents or legal representatives prior to the study.

This research has been approved by the Medical Ethical Commission of the VU University Medical Centre Amsterdam as well as the VU University Science and Ethics board.

### Medication

Nine participants use medication (Zomacton, Movicolon, Ventolin, Zyrtec, Aerius, D-Amo-X.Z., Broxil, and Flixotide), however these were not excluded from testing (medication had to the authors' knowledge no influence on the administered tests).

### Materials

Participants were matched for inclusion into statistical analysis using variables from our intake-questionnaire. Matching criteria obtained from the questionnaires were parents' socio-economic status, medication, and prior medical issues as well as exposure to a musically enriched environment. Additionally, identification of strengths and weaknesses of participants (strengths and difficulties questionnaire SDQ-Dut; Goodman, 2001), reward and punishment sensitivity in children with ADHD SPSRQ-C (Goodman, 2001), possible traits of high-functioning autism (Autism Quotient for children; Auyeung et al., 2008), and ADHD (Strengths and weakness of ADHD-symptoms and normal behavior, SWAN; Luman et al., 2008), formed part of this intake questionnaire. These were added to ensure the results were not skewed by possible pathologies.

Furthermore, the socio-economic background of participants was assessed by the highest education level of both parents, whereby all parents have scored above secondary school level.

Participants were followed over a period of 2.5 years and identical neuropsychological tests were administered every 6 months (T0 – T4). To minimize a possible learning effect, the tests increased progressively in difficulty e.g., increase in items to be remembered or moved (see details under specific test).

### Neuropsychological test battery

The analysis of executive functions covers a wide range of such functions. Our focus was on planning, inhibition, short term memory, and working memory. All of these tests were administered by using an iPad 2 or 3 running iOS 7 or higher (screen size 9.7 inch, 1,024 × 768, 132 ppi, multitouch) minimizing researcher bias. These tests represent the traditional “pen and paper” and “manual tests” and were coded by programming specialists and the researchers in Apple script with Apple Developer Xcode 6 software.

All tests, with exception of the Tower of London (see sub-section ToL), were scored according to the validation and scoring criteria as stated in each testing manual or handbook. These criteria were translated into the computerized versions used here. All output data use customized algorithms to present results in final scores per test, test trial, and participant.

### Planning: tower of london (ToL)

Planning was tested with the Tower of London test (ToL) (Shallice, 1982), which was shown to be a valid measure of higher order problem solving. Participants performed several tasks by sorting colored balls to match the provided final constellation of the balls in as few moves as possible. Balls were shifted on three rods and could only be moved from rod to rod one at a time. Time to complete the task, as well as number of moves to achieve the goal, were measured. Test-retest reliability of the ToL was adequate at *r* = 0.739 and 0.734 (Schumacher et al., 2015).

As there is no suitable scoring method for the Tower of London in the literature, which allowed more subtle differences in scoring—rather than simply correct or incorrect—the authors comprised a refined scoring methodology (see Appendix I in Supplementary Material).

### Visuospatial short term and working memory: klingberg short term and working memory task

Visuospatial working memory and short-term memory was measured using a dot matrix, whereby participants were required to select dots appearing in a four by four grid in forward and reverse order (Alloway, 2007; Dumontheil and Klingberg, 2012). The task involved remembering the location of the dots. Difficulty increased through adding additional dots up to a maximum of seven as well as changes in location of the dots. Test-retest validity and reliability was highly correlated with the components span forward and span backward of the test, *r* = 0.79 (Waters and Caplan, 2003; Sung, 2011). Each level represented four trials with increasing number of dots (Level 1 = 4 × 2, Level 2 = 4 × 3, Level 3 = 4 × 4, Level 5 = 4 × 5, and Level 6 = 4 × 6). Multiplying the correct trials times the level reached was included into the algorithm, as our version divided the total amount of trials with the overall reaction time. This number however, did not represent a correct value and had to be multiplied with the amount of levels to correct for the total reaction time resulting in a corrected final value for each participant.

### Inhibition: go/no-go task

Inhibition was assessed with a go /no go paradigm (Nosek and Banaji, 2001; Lakatos et al., 2013), which measured the ability to inhibit a motor response to a presented visual stimulus. In one outcome, the participant was required to perform a response (go condition) and withhold a motor response when the object was crossed through (no go condition). Accuracy and reaction time were measured to give an indication of the participants' inhibitive qualities.

The version used here was created specifically for children and depicts a plane, which either flies left or right. If the plane is crossed through, the respondent should not press the left or right button on the screen. Difficulty was increased progressively adding a latency time of 10 ms (cross appears later than the plane). The better the child performs, the later the cross appears. Equally, if the participant presses before the cross appears (performing worse on the no-go stimulus) the latency time diminished and the cross was added earlier after the plane appears. Test-retest coefficients were satisfactory for the stop task Mean probability of inhibition *r* = 0.72, Mean reaction time MRT *r* = 0.66, Total Errors *r* = 0.49, Slope of inhibition function *r* = 0.32 and standardized Stop Signal Reaction Time SSRT *r* = 0.21. To determine the final stop signal reaction time, the total amount of stop signal delay time (as delay times vary per trial, the amount of delay was different per participant amounting to an average stop signal delay time) was subtracted from the mean reaction time (MRT) divided by the percentage of errors. SSDT subtracted from MRT resulted in a raw value which could be corrected for amount of errors made, resulting in a standardized Stop Signal Reaction Time. The level of inhibition was determined through an error percentage corrected for *standardized Stop Signal Reaction Time* (SSRT), whereby a lower SSRT indicated a better ability to inhibit during the stop stimulus. We opted for this approach as a more conservative scoring may have reflected high accuracy scores, however low processing speed resulted in low efficiency scores(Logan and Cowan, 1984; Votruba and Langenecker, 2013).

### Verbal IQ

The Wechsler Intelligence Scale for Children 3rd Edition subtests indicate the child's IQ; Sub-tests I (Information), III (Similarities), V (Verbal Comprehension), and VII (Arithmetic) were administered (Wechsler, 1991).

WISC-III-NL shortened version is based on acceptable reliability and validity on subtests vocabulary (α = 0.96, *r* = 0.85), similarities (α = 0.93, *r* = 0.81), arithmetic (α = 0.93, *r* = 0.74), and information (α = 0.95, *r* = 0.82). The combination of these subtests showed highest internal consistency according to the split-half method and highest correlations with the total verbal IQ scale in comparison with the other subtests of the WISC-III-NL (Wechsler, 1991).

Verbal IQ was chosen over a whole scale or a performative IQ as education methods in Dutch primary schools are based on verbal problem solving in both language and arithmetic.

### National pupil monitoring system (CITO)

Academic performance was assessed using the pupil's performance on tests from the Dutch National Pupil Monitoring System (van Delden, 1994). This system has been used throughout 80% of all Dutch primary schools in order to keep track of the pupil's self- and peer-referenced academic development throughout their education (Vlug, 1997). Tests from the Dutch National Pupil Monitoring System are administered during three testing periods throughout each academic year.

CITO tests measure abilities such as different language skills, listening, spelling (A), writing, vocabulary (B), decoding (C), reading comprehension (D), and arithmetic (E) according to the item-response model (van Delden, 1994; Vlug, 1997). These tests have been evaluated by the Dutch Committee on Tests and Test Affairs (COTAN) and are considered to have adequate test validity, reliability and norms (COTAN Arnhem The Netherlands, 1996, 1998, 2002, 2010). The norm scores (A–E) were used to determine academic achievement.

### Music intervention

Our music intervention has been developed in collaboration with the Ministry of Research and Education in the Netherlands and an expert center for arts based education (MOCCA). This intervention was designed for all primary schools in the Netherlands. Of note, not all primary schools receive this curriculum yet, it is however the aim to provide all Dutch primary schools with this intervention by the year 2020.

Early lessons introduced melody, meter and rhythm as well as the different instruments in both classical and popular music settings. Lessons were additionally designed around the knowledge of the basic fundamentals of music and were part of a structured curriculum designed by expert centers in education. Children were encouraged to choose and play instruments. Theoretical lessons were given by trained music teachers for primary schools. Active instrumental lessons were supervised by trained music teachers and performed in class. Children in the Music group did not take the instruments home. Participants in the music group followed this program in a structured manner, receiving 1–2 h lessons weekly during regular school hours. A regular lesson starts with a welcoming song, followed by music theoretical and historical information in the context of the song and ends with collective music making, singing and improvising. Children therefore learn to listen, play, and improvise.

### Visual arts

This intervention was developed by the consortium of fine arts educators in the Netherlands. The Visual Arts group received general lessons in painting, sculpting and arts history. This intervention, equally as the music intervention is supposed to be applied throughout all Dutch primary schools by the year 2020. The focus was on the practical application of skills contributing to the creation of visual art. Fully trained arts teachers gave both theoretical and practical classes. Children in the visual arts group were painting, sculpting and using different materials to create artworks as well as learning about art history.

### Control

The control group did not receive any arts lessons in addition to the usual curriculum. However, participants in the control group were likely to be painting and singing songs as it is a primary school setting, but not as part of the curriculum or a structured educational program.

### Procedure

Trained research assistants administered each test. Participants were tested individually in a quiet environment during school hours. Total testing time was 1.5 h per participant minimizing disturbance during school hours as much as possible.

**Figure 1:**
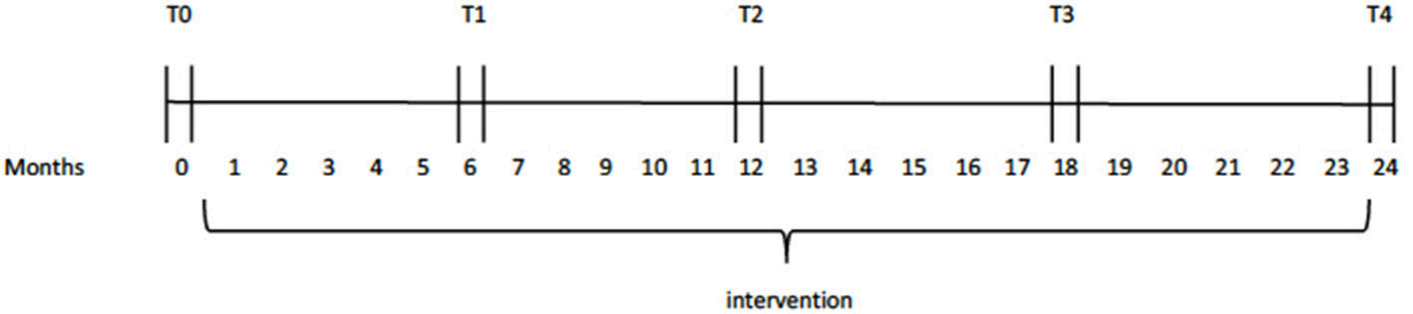
Schematic representation of testing moments (T).

The whole test protocol was administered in one session with short breaks, where necessary, to motivate and allow the participants to regain their focus on the tasks. All tasks were presented in a child-friendly manner and aimed at creating a “computer-game” environment. The protocol was administered to both the experimental as well as the control groups in five stages: pre-intervention measurement (baseline measures); 1st follow up; 2nd follow up; 3rd follow up and final follow up. Participants were followed for two-and-a-half years, with each testing moment (T0 – T4) 6 months apart (Figure 1).

### Statistical analysis

As we expected a significant effect over time and recorded a low amount of missing values, a Multiple Mixed-Sample Repeated Measures Analysis of Covariance (split-plots ANCOVA) was used to identify effects of music education on cognitive development per condition over time with age as covariate. Additionally, contrast analyses computed comparisons of groups per test per testing moment. As is suggested by Howell (2010), a split-plot or mixed design analysis of (co)variance analyses was chosen as we have block-randomized our participants. A split-plot design, therefore, applies experimental factors across a block randomized form, allowing calculation of sub-unit effects (EF) within the application (here music intervention).

Data from the baseline measurement (T0) and four follow-ups (T1, T2, T3, and T4) were included in the analyses. Descriptive statistics were computed for the overall scores of the tests, including intercorrelations. Normality and homogeneity were analyzed using the Levene's test.

Pair-wise *post-hoc* analysis compared groups per measurement per moment in time to indicate differences between conditions using the Bonferroni confidence interval adjustment.

The split-plots ANCOVA was set up as 4 (condition) × 5 (time), whereby condition represents either group (MUSIC, MUSIC+, VISUAL ARTS, and NO ARTS EDUCATION) and time represents the measurement (T0 – T4).

### Mediation analysis

The Sobel test was calculated for each possible condition × time to analyse results from neuropsychological test scores to scores on the national pupil monitor (CITO) (Preacher and Hayes, 2004, 2008).

**Figure 2:**
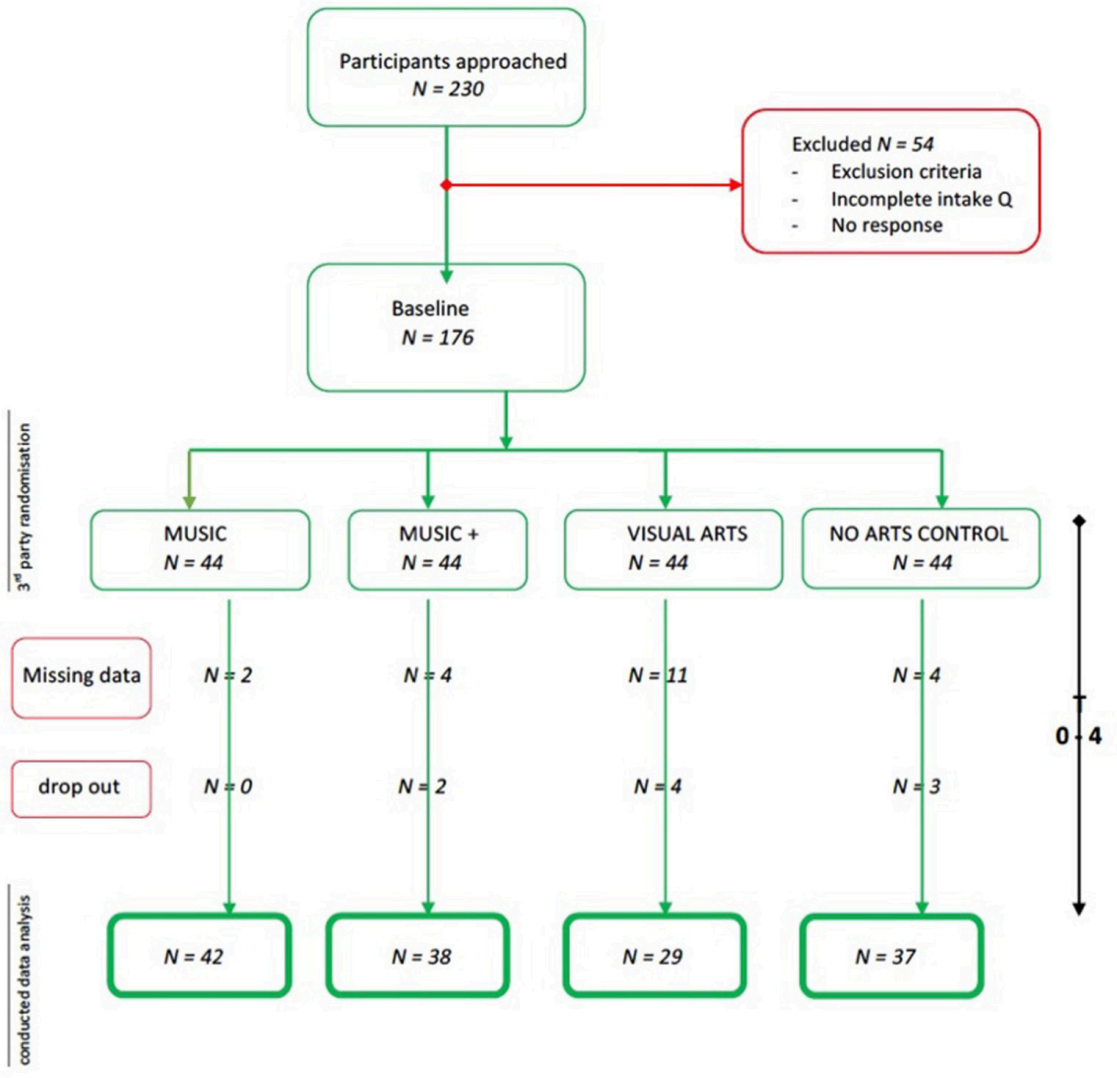
Participant flow chart.

Participants with missing values have been excluded from the analysis (Figure 2).

To perform the above-mentioned analysis, SPSS Statistics and R statistical software were used (SPSS 24, IBM and R Language 3.3.2.). Level of significance was set at *p* < 0.05.

## Results

### General

An initial chi-square analysis revealed no significant differences between the four groups in gender distribution, $\chi_{\left(2\right)}^{2}$ = 0.47, *p* = 0.79.

Ages were grouped per grade (grade 3–4 at T0 and T1; age 6–8, grade 5–6 at T2 and T3; age 7–9 and grade 7–8 at T4; age 8–10) and a chi-square analysis has shown no significant difference between the age groups across time $\chi_{\left(5\right)}^{2}$ = 1.57, *p* = 0.844.

Levene's test indicated that the variances were equal for the four groups at baseline, *F*_(2, 147)_ = 0.87, *p* = 0.42, first follow-up, *F*_(2, 147)_ = 0.85, *p* = 0.43, second follow-up, *F*_(2, 147)_ = 2.83, *p* = 0.06, third follow-up, *F*_(2, 147)_ = 0.44, *p* = 0.64, and forth follow-up *F*_(2, 147)_ = 1.75, *p* = 0.42, meaning equal distribution of the sample per testing moment.

Additionally, the participants socio-economic background, as measured by the mean of highest parental educational level, revealed equal distribution across the four groups $\chi_{\left(4\right)}^{2}$ = 0.39, *p* = 0.75; MUSIC *M*_mother_ = 6.08, *SD* = 1.01, *M*_father_ = 6.01, *SD* = 0.98; MUSIC + *M*_mother_ = 5.99, *SD* = 1.02, *M*_father_ = 6.05, *SD* = 0.98; VISUAL ARTS *M*_mother_ = 6.07, *SD* = 0.99, *M*_father_ = 5.98, *SD* = 0.95; and NO ARTS CONTROL *M*_mother_ = 6.00, *SD* = 1.24, *M*_father_ = 6.03, *SD* = 0.96.

Table 1 summarizes descriptive statistics of each test per group. A split-plot ANCOVA with additional contrast analysis was computed for each group showing an overall effect of group (both music groups vs. both controls) and Group × Time (individual groups × overall T) as well as a comparison of groups per test per testing moment. Assumptions for a split-plot approach and ANCOVA were met.

**Table 1:** Describes descriptive statistic showing mean scores and standard deviation (SD) are shown for each group in time (T0–T4) across all administered tests.

	**MUSIC**					**MUSIC +**					**FINE ARTS**					**NO ARTS CONTROL**				
	**WISC**	**WM_*F*_**	**WM_*B*_**	**INH**	**PLAN**	**WISC**	**WM_*F*_**	**WM_*B*_**	**INH**	**PLAN**	**WISC**	**WM_*F*_**	**WM_*B*_**	**INH**	**PLAN**	**WISC**	**WM_*F*_**	**WM_*B*_**	**INH**	**PLAN**
T0	111.8 *(2.474)*	7.46 *(3.257)*	5.46 *(3.378)*	0.423 *(0.287)*	24.1 *(2.651)*	110.9 *(3.118)*	7.34 *(3.497)*	4.97 *(2.662)*	0.438 *(0.239)*	26.5 *(2.124)*	111.3 *(2.649)*	7.32 *(2.539)*	5.64 *(3.058)*	0.502 *(0.259)*	28.1 *(2.326)*	111.5 *(2.815)*	7.21 *(3.373)*	5.85 *(3.321)*	0.509 *(0.269)*	27.9 *(2.366)*
T1	114.2 *(3.190)*	8.52 *(3.684)*	6.17 *(3.131)*	0.418 *(0.225)*	26.4 *(2.354)*	116.1 *(2.311)*	8.71 *(3.326)*	7.29 *(2.929)*	0.369 *(0.207)*	27.1 *(2.842)*	115.7 *(3.077)*	8.45 *(3.191)*	7.31 *(2.965)*	0.405 *(0.288)*	28.9 *(3.215)*	110.8 *(2.502)*	9.55 *(3.236)*	6.66 *(2.623)*	0.398 *(0.254)*	28.2 *(3.758)*
T2	118.9 *(2.697)*	10.07 *(3.595)*	6.45 *(4.248)*	0.335 *(0.165)*	32.6 *(3.956)*	116.0 *(2.296)*	10.26 *(3.222)*	7.43 *(3.666)*	0.436 *(0.265)*	33.5 *(3.001)*	114.3 *(2.352)*	8.47 *(2.776)*	7.32 *(3.845)*	0.432 *(0.265)*	32.2 *(3.398)*	109.0 *(2.894)*	7.96 *(3.588)*	7.40 *(3.651)*	0.494 *(0.251)*	30.9 *(2.653)*
T3	117.9 *(3.067)*	10.32 *(3.652)*	7.78 *(3.384)*	0.327 *(0.222)*	34.1 *(2.998)*	117.5 *(2.202)*	10.57 *(3.702)*	8.47 *(4.142)*	0.337 *(0.261)*	35.1 *(2.481)*	114.4 *(3.726)*	10.89 *(2.805)*	8.33 *(2.473)*	0.457 *(0.261)*	32.8 *(2.864)*	110.1 *(3.41)*	10.59 *(4.193)*	7.79 *(4.029)*	0.475 *(0.261)*	32.9 *(3.322)*
T4	123.0 *(2.946)*	11.17 *(4.025)*	7.98 *(4.419)*	0.315 *(0.198)*	36.2 *(2.845)*	120.5 *(3.045)*	11.67 *(4.580)*	7.97 *(4.254)*	0.319 *(0.223)*	35.9 *(3.201)*	118.1 *(2.655)*	11.00 *(2.625)*	9.25 *(3.273)*	0.479 *(0.256)*	33.1 *(3.985)*	112.1 *(2.920)*	10.51 *(3.592)*	7.66 *(4.242)*	0.479 *(0.239)*	33.9 *(3.256)*

A subsequent comparison of means indicated no significant differences at baseline across the four groups.

### Visuospatial working memory

**Figure 3:**
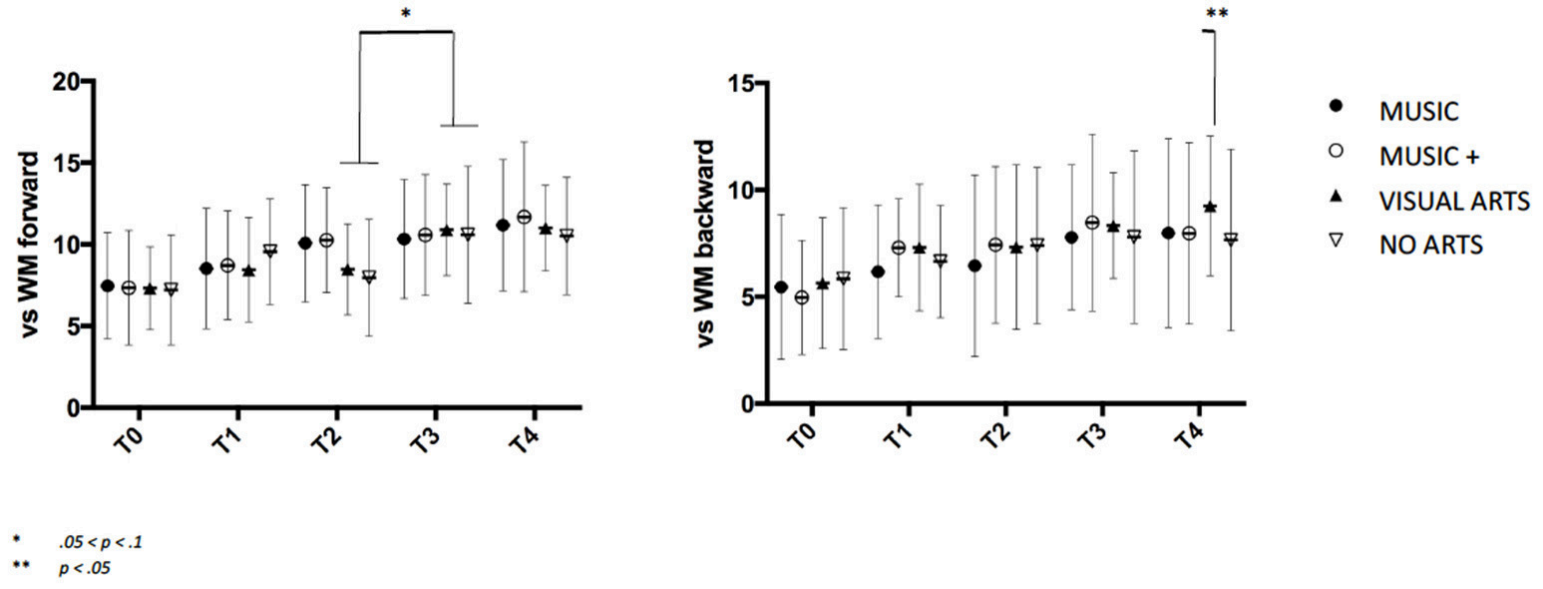
Visuospatial working memory span over time with standard deviation. The left graphs show the differences of all four groups on the visuospatial sketchpad, the right graph shows increase on the central executive.

A significant Group × Time interaction was found on the visuospatial sketchpad^4^ for the VISUAL ARTS group, WM_Forward_ *F*_*F*__(2, 147)_ = 4.061, *p* < 0.01 and the central executive WM_Backward_ *F*_*B*__(2, 147)_ = 4.455, *p* < 0.05. Overall effect of Group was significant at *F*_(2, 146)_ = 5.165, *p* < 0.05. Even though all four groups show an increase in mean scores (Table 1), contrast analysis has shown the VISUAL ARTS group increased significantly on the central executive at T4 as compared to the no arts control *F*_(2, 146)_ = 2.353, *p* = 0.03 and both music groups MUSIC *F*_(2, 145)_ = 3.546, *p* < 0.05 and MUSIC + *F*_(2, 145)_ = 4.854, *p* < 0.05 (Figure 3).

### Verbal intelligence

**Figure 4:**
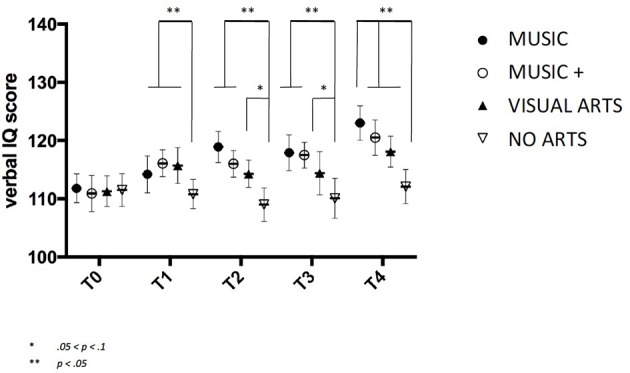
Verbal intelligence score over time with standard deviation.

All four groups have increased mean values on the verbal intelligence measure (Table 1). More so, the verbal intelligence test revealed a Group x Time interaction *for both MUSIC F*_(2, 145)_ = 3.465, *p* = 0.041 and MUSIC + *F*_(2, 146)_ = 2.952, *p* < 0.05 and VISUAL ARTS *F*_(2, 146)_ = 2.743, *p* = 0.05 as well as an overall effect of Group *F*_(2, 147)_ = 4.48, *p* < 0.01. Contrast analyses revealed an overall significant increase of verbal IQ from baseline to T4 in both MUSIC *F*_(2, 146)_ = 5.11, *p* < 0.05 and MUSIC + *F*_(2, 146)_ = 4.984, *p* < 0.05 as compared to both controls (Figure 4).

### Planning

The ability to plan and execute a task mentally and practically yielded a Group × Time interaction MUSIC *F*_(2, 145)_ = 3.189, *p* = 0.05 and MUSIC + *F*_(2, 145)_ = 3.485, *p* < 0.05.

**Figure 5:**
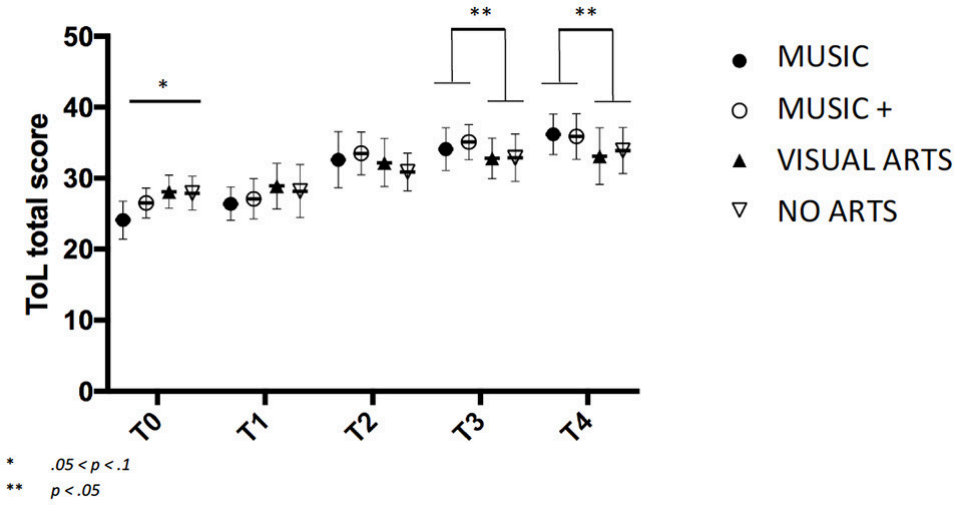
Increase on the total score obtained in the Tower of London task with standard deviation.

Overall effect of Group was significant at *F*_(2, 147)_ = 4.177, *p* = 0.05. Both MUSIC groups have increased significantly on planning ability from T2 to T3 and T3 to T4 as compared to the no arts control through contrast analysis *F*_*T*2−3__(2, 147)_ = 2.353, *p* = 0.03 and *F*_*T*3−4__(2, 146)_ = 2.112, *p* = 0.05 or the VISUAL ARTS group *F*_*T*2−3__(2, 145)_ = 2.845, *p* < 0.05 and *F*_*T*3−4__(2, 146)_ = 2.165, *p* < 0.05 (Figure 5).

### Inhibition

**Figure 6:**
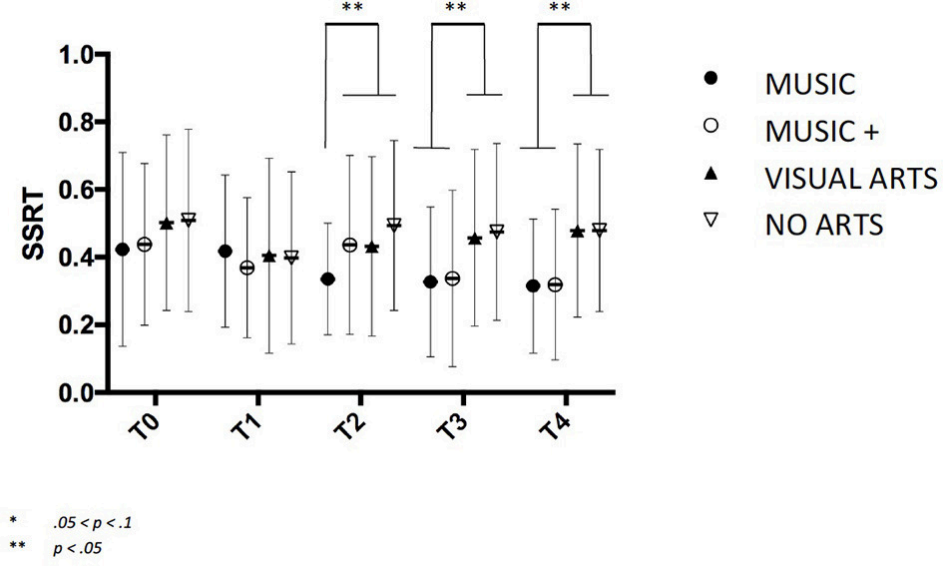
Inhibition levels as measured on stop stimulus reaction time. A lower SSRT indicates a more efficient level of inhibition with standard deviation.

A significant Group × Time interaction was found on the standardized stop signal reaction time value for MUSIC *F*_(2, 147)_ = 2.114, *p* < 0.05, and MUSIC + *F*_(2, 147)_ = 2.955, *p* < 0.05 as well as an overall effect of Group *F*_(2, 146)_ = 3.678, *p* = 0.05. Contrast analysis has shown significant decrease of SSRT scores from T2 to T3 to T4 for both MUSIC *F*_*T*2−3__(2, 145)_ = 4.455, *p* < 0.05, *F*_*T*3−4__(2, 145)_ = 3.497, *p* = 0.05 and MUSIC + *F*_*T*2−3__(2, 145)_ = 4.144, *p* < 0.05, *F*_*T*3−4__(2, 145)_ = 4.014, *p* < 0.05 (Figure 6).

### Verbal IQ, planning, and inhibition scores as mediator

Sobel mediation analyses were conducted to examine whether the increase of verbal IQ, planning and inhibition scores could explain an increase in overall CITO scores for the music groups, thus implying a possible far transfer effect. The analysis revealed that children in either music condition and in the visual arts condition did significantly differ in average performances on CITO scores at T4 as compared to the NO ARTS control, through contrast analysis MUSIC *F*_(2, 147)_ = 3.658, *p* < 0.05, MUSIC + *F*_(2, 147)_ = 3.145, *p* < 0.05, VISUAL ARTS *F*_(2, 147)_ = 3.712, *p* = 0.05 as well as Group × Time MUSIC *F*_(2, 146)_ = 2.735, *p* < 0.05, MUSIC + *F*_(2, 146)_ = 2.984, *p* = 0.05, VISUAL ARTS *F*_(2, 146)_ = 2.577, *p* = 0.05. Subsequent comparison of means did not indicate a significant difference between groups at baseline. Overall Group effect yielded *F*_(2, 145)_ = 5.973, *p* < 0.05. Furthermore, using verbal IQ, planning and inhibition as mediators in revealing a possible effect from music education to the national pupil monitoring scores, the investigated executive sub-functions have shown a positive Sobel test mediation effect for MUSIC *t*_IQ_ = 13.54, *p* = 0.05; *t*_INH_ = 11.35, *p* = 0.05; *t*_PLA_ = 12.75, *p* = 0.04 and MUSIC + *t*_IQ_ = 12.94, *p* < 0.05; *t*_INH_ = 11.78, *p* < 0.05; *t*_PLA_ = 13.01, *p* = 0.05 suggesting far transfer from music education to academic achievement as measured by a centralized monitor.

## Discussion

The goal of the present study was to examine whether structured music lessons can affect executive sub-functions that may underlie academic achievement. The results show that children following structured music lessons perform better on tasks measuring verbal IQ, planning and inhibition when compared to controls during four follow ups.

Participants have been matched on parents' socio-economic status, medication and prior medical issues as well as exposure to a musically enriched environment and private music lessons. Overall effect of Group, as well as Group × Time effects, were significant for the MUSIC and MUSIC + group when compared to the VISUAL ARTS group and NO ARTS control on verbal intelligence, planning and inhibition. Additionally, the VISUAL ARTS group improved significantly on visuospatial memory. Ho et al. (2003), have previously argued similar results, by concluding that musical training did not improve visual memory. The authors however did not compare the music group to a visual arts group. Practicing visual arts engages neural networks, which overlap the representation of imagery and working memory in a three-dimensional space (Winner and Drake, 2013). Our VISUAL ARTS group followed a structured two-and-a-half-year long program in visual arts, which therefore, can promote neuroplasticity in the domain of visuospatial working memory as compared to a non-arts or sole music intervention (Dulamea and Dulamea, 2011).

Arguing executive sub-functions as paramount for the perception, processing, and execution of music, our study has found significant increases on inhibition and planning as well as verbal IQ after two-and-a-half years of music intervention. Against this backdrop, computerized music programs, lasting only 20 days, have also shown increased performance on inhibition in school-aged children (Moreno et al., 2011). Our music program has used a more traditional approach; teaching children to play an instrument, sing actively and listen to music in class, which is in contrast with mainly listening in the computerized interventions. Even though we found a positive effect of long-term music lessons to inhibition, Moreno et al.'s (2011) event-related potential recordings might be much more sensitive to short-term changes in inhibitory control. Nevertheless, our neuropsychological test battery has shown significant improvement of inhibition levels in both music groups. Improvement in executive sub-functions can be explained through research promoting neural plasticity via long-term interventions (Schlaug et al., 2009; Dulamea and Dulamea, 2011; Winner and Drake, 2013; Schlaug, 2015). Researchers have shown that practicing music for a longer period of time increases connectivity of the corpus callosum thus strengthening communication between both hemispheres and, more so, appealing to connectivity in the ventro-lateral (VL-PFC) and medial prefrontal cortex (M-PFC) (Zuk et al., 2015). As a *by-product*, overlapping prefrontal cortex structures associated with inhibition and planning in those networks also improve (Zuk et al., 2015). Executive sub-functions such as planning, inhibition and working memory are thus equally recruited while playing music as when solving an arithmetic problem (Zuk et al., 2015). It is this delicate balance between music training and executive sub-functions which serve as mediator to academic achievement. Even though we have not used brain imaging in our study, the used neuropsychological test battery has indicated an increase in inhibition, planning and working memory, all three of which are associated with ventro-lateral and medial prefrontal cortex activity (Zuk et al., 2015). Contradictory to these results, Zuk et al. (2015), did not observe a difference in inhibition levels in their sample of 27 children. The authors argue that more careful subject selection, matching and the sample size may explain differences in inhibitory performance between the music and non-music group. Furthermore, it is difficult to interpret their results as the music group had different levels of musical training and musical aptitude could not be excluded (Zuk et al., 2015). In contrast, our study has offered a structured and standardized music curriculum to the music groups as part of the regular school curriculum reaching every participating pupil, therefore minimizing possible motivational factors. A structured music curriculum, meaning increasing in difficulty as the children improve on the musical tasks, approaches music education from a more pedagogical angle. While studies have generally used basic music programs, such as singing together or clapping (Mehr et al., 2013), developing a standardized music curriculum for primary schools together with the expertise of the ministry of education and an expert center for arts-based education, amalgamates knowledge from education, application and general skill development of young children (Hetland and Winner, 2004; Winner, 2011). Structured and standardized programmes clearly define student input and learning outcomes, which are central to regular education, and need to be equally applied to music education. This stands in juxtaposition to the commonly developed and used musical interventions for the sole purpose of researching their effects on cognition or academic skills, seldom continuing these interventions once the investigation is finalized. A structured music curriculum therefore, places the student at the center of music education, investing in the development of musical skills across the pupil span.

All factors combined, such long-term investments into education together with our results support the claim that long-term music interventions improve academic achievement and executive sub-functions, such as inhibition and planning, as measured with neuropsychological tests. Even though more studies investigating executive sub-functions, on the relation between music education and academic achievement are necessary, our study has attempted to close this gap.

## Limitations

One limitation in this study is the generalized student monitor system. Even though it scores students on individual tasks such as critical listening or writing, we did not compare the scores of the CITO to other tests that, for example, measure phonological awareness in the context of writing or critical listening. Comparing the CITO scores, which are a generalized *special* model for Dutch children, with more internationally standardized language functioning tests (e.g., Phonological assessment battery; Gallagher and Frederickson, 1995), may yield a more in-depth view of phonological skills, writing, or reading. However, this approach would increase testing time to more than 3 h, taking all academic skills with all their sub-components into consideration (e.g., phonological awareness, semantics and lexemes in language and logic, abstract thinking, and computation in mathematics). Moreover, longer testing times can influence overall disturbance and concentration of participants. Additionally, this study has focussed on verbal IQ alone, not administering a full IQ scale, which might possibly show a different result on intelligence measures (Schellenberg, 2006; Dumont et al., 2017).

## Conclusion

Executive functions are usually researched as *lump sum* cognitive functions and structured investigation of sub-functions in longitudinal designs are still rare. The here presented results show a possible far transfer effect from a structured music education program to academic achievement, mediated through executive sub-functions. Nevertheless, analyzing the longitudinal effects of music education embedded into the regular school curriculum, throughout different cultural settings, will further strengthen our understanding of the effects music can have on the developing brain.

In the end, it is not the justification of music or arts education in light of far transfer to academic achievement that is the objective. It is the necessity of combining music, visual and general arts toward a mixed-art education model. This will emphasize the importance of the arts in human culture, and enduringly support the positive influence of the arts on cognitive development.

## Author contributions

AJ, HH, and ES have equally contributed to the conception and design of the work. Data collection as well as analysis and interpretation have been supervised and executed by AJ. The corresponding author has drafted and revised the manuscript with critical revisions and final approval for publication by both HH and ES.

### Conflict of interest statement

The authors declare that the research was conducted in the absence of any commercial or financial relationships that could be construed as a potential conflict of interest.

## Footnotes

^1^Representing *N* = 230 pupils in total, sample size calculation with sufficient statistical power; G^*^Power version 3.1.2, *a* = 0.50, *b* = 0.80, calculated *N* = 117, recruited *N* = 230 and *N* = 176 tested at baseline.

^2^Descriptive statistics are described in the general Results section.

^3^e.g., was extremely high or extremely low and thus be classified as either a system error or for example IQ values of above 170 points, whereas the maximum IQ score can reach only 160 points for that age.

^4^*WM f* = *Short term Memory & WM b* = *Working Memory*
