# Supplementary material for: Longitudinal Analysis of Music Education on Executive Functions in Primary School Children
Source: Front Neurosci. 2018 Feb 28;12:103. doi: 10.3389/fnins.2018.00103 (PMC5835523; doi:10.3389/fnins.2018.00103)
Supplement: Supplementary file 1 [file DataSheet1.DOCX]

**Appendix I**

*Tower of London scoring*

This score is based on the maximum points to be obtained per trial, respectively one point for the three-ball problem, two points for the four-ball problem and three points for the five and six ball problem.

| Minimum moves | Max points^21^ |
| --- | --- |
| (Problem 1 = 4 x 3) 3 | 1 |
| (Problem 2 = 5 x 4) 4 | 2 |
| (Problem 3 = 5 x 5) 5 | 3 |
| (Problem 4 = 5 x 6) 6 | 3 |
| Minimum moves for all trials: 87 | Maximum points for all trials: 44 |

Each excess move (XS moves which are more than the minimum moves necessary to solve the problem), are subtracted from one another and multiplied by ten to yield an excess error percentage.

XS_moves_ - MIN_moves_ = XS_score_

- XS_score_ x 10

= % XS_score_

This percentage is subtracted from the maximum amount of points to be obtained per problem, yielding the overall amount of points, between 0.0 and depending on the trial 1.0/2.0/3.0.

MAX_points_ - % XS_score_

= ToL_score/trial_

The sum of all trials yields the final score, which can reach a maximum of forty-four points.

∑ ToL_score/trial_

= ToL_points_

Points can reach on equal distances from 0.0 to 40.0, with stepwise .1 increase or decrease.
